# Manipulation of Miniature and Microminiature Bodies on a Harmonically Oscillating Platform by Controlling Dry Friction

**DOI:** 10.3390/mi12091087

**Published:** 2021-09-09

**Authors:** Sigitas Kilikevičius, Algimantas Fedaravičius, Virginija Daukantienė, Kristina Liutkauskienė, Linas Paukštaitis

**Affiliations:** 1 Department of Transport Engineering, Kaunas University of Technology, Studentų St. 56, 51424 Kaunas, Lithuania; algimantas.fedaravicius@ktu.lt (A.F.); virginija.daukantiene@ktu.lt (V.D.); kristina.liutkauskiene@ktu.lt (K.L.); 2 Department of Energy, Kaunas University of Technology, Studentų St. 56, 51424 Kaunas, Lithuania; linas.paukstaitis@ktu.lt

**Keywords:** manipulation, dry friction, control, vibrations, harmonic oscillations, platform

## Abstract

Currently used nonprehensile manipulation systems that are based on vibrational techniques employ temporal (vibrational) asymmetry, spatial asymmetry, or force asymmetry to provide and control a directional motion of a body. This paper presents a novel method of nonprehensile manipulation of miniature and microminiature bodies on a harmonically oscillating platform by creating a frictional asymmetry through dynamic dry friction control. To theoretically verify the feasibility of the method and to determine the control parameters that define the motion characteristics, a mathematical model was developed, and modeling was carried out. Experimental setups for miniature and microminiature bodies were developed for nonprehensile manipulation by dry friction control, and manipulation experiments were carried out to experimentally verify the feasibility of the proposed method and theoretical findings. By revealing how characteristic control parameters influence the direction and velocity, the modeling results theoretically verified the feasibility of the proposed method. The experimental investigation verified that the proposed method is technically feasible and can be applied in practice, as well as confirmed the theoretical findings that the velocity and direction of the body can be controlled by changing the parameters of the function for dynamic dry friction control. The presented research enriches the classical theories of manipulation methods on vibrating plates and platforms, as well as the presented results, are relevant for industries dealing with feeding, assembling, or manipulation of miniature and microminiature bodies.

## 1. Introduction

The proliferation of miniature products in mass production has led to the development of microtechnologies and related sciences. The efficiency of manipulation and assembly of miniature and microminiature components currently are of great concern to various industries related to microtechnologies. For manipulation of miniature and microminiature bodies, a variety of techniques are used, ranging from prehensile pick and place techniques [1,2,3,4,5,6] to nonprehensile methods such as vibration-assisted manipulation [7,8,9]. However, prehensile techniques such as picking always have some mechanical effect on the body; besides, due to various shapes of the miniature and microminiature bodies being manipulated or assembled, microgrippers must be changed frequently when pick and place operations are used. In addition, these methods still struggle with precise force feedback at the micro scales and are suited for manipulations with individual bodies.

Various micropositioning platforms have been investigated for microminiature bodies as an alternative. Ferrara-Bello et al. [10] proposed a micropositioning platform actuated by three piezoelectric stacks intended to control the displacements along the x, y, and z axes. Three structural materials (polylactic acid, acrylonitrile butadiene styrene, and polyethylene terephthalate glycol) were used for manufacturing the proposed micropositioner. It can be integrated into a microgripper to have a complete manipulation system that delivers larger displacements than most other commercial solutions. However, the developed piezo stacks generated much lower forces. Guo et al. [11] designed a compact planar flexure-based mechanism for micropositioning in three degrees of freedom, which was monolithically manufactured using wire electro-discharge machining. In order to reduce the nonlinear hysteresis and external disturbance of the flexure-based mechanism, a hybrid feedforward/feedback controller was also proposed. Sun et al. [12] proposed an equal-stiffness and equal-stroke 2D micropositioning small size platform driven by piezoelectric actuators adopting two hourglass displacement magnification mechanisms based on the static stiffness analysis and simulation analysis of the micropositioning platform. Al-Jodah et al. [13] proposed a compact mechanism for micro/nanopositioning platforms that combines voice coil motors and piezoelectric actuators. The proposed design, along with the improved control method, was capable of achieving a large workspace and a high motion accuracy. Li et al. [14] proposed a flexure-based system for nanopositioning with dynamically tunable properties by an external magnetic field. This is implemented through magnetorheological elastomers placed around the flexure beams of the system. However, methods that employ micromanipulation platforms do not provide large workspaces.

The use of vibration-assisted and acoustic methods for micromanipulation has been prevalent recently [15]. These methods are relatively easily implemented and cost-effective techniques, especially for manipulation with high quantities of miniature and microminiature particles, as they do not require grippers and there is no mechanical contact or contamination involved [16]. Near-field acoustic manipulation, which is suitable to transfer micro and nanoparticles in a confined evanescent Bessel beam, was proposed by Gires and Poulain [17]. Wijaya et al. [18] analyzed the manipulation of microspheres by applying acoustic levitation. The micromanipulation device was composed of a centrally actuated vibrating platform and a reflector. It was demonstrated that the frequency and the tilt angle between the plate and the reflector define the position of microspheres. Fakhfouri et al. [19] analyzed manipulation with particles and cells by employing traveling surface acoustic waves in a microfluidic system. They determined the influence of the size of particles on their behavior as a function of both acoustic wavelength and power. They also identified three different forcing mechanisms: swirling due to the acoustic streaming forces, migration due to the acoustic radiation forces, and patterning due to the diffraction effects. The acoustic radiation force was exploited by Xu et al. [20] for acoustic manipulation of microparticles in a cylindrical cavity.

Vibration-assisted methods exploit horizontal or vertical (and sometimes a combination of both) vibrations to deliver particles along the desired direction at a certain velocity. These methods provide large workspaces and low operational times. An asymmetry is a necessary condition to make a body move on a vibrating platform, which ensures that net friction forces over one oscillation cycle do not cancel out. Reznik et al. [21] investigated the motion of a body on a vibrating platform, which was subjected to an asymmetric excitation. The temporal asymmetry (also called vibrational or time-asymmetry) resulted in the motion of bodies along a straight-line path. Kumar and DasGupta [22] excited circumferential harmonic traveling waves on a thin circular plate to be used for manipulation of microminiature particles. Viswarupachari et al. [23] investigated the manipulation of particles over a flat horizontal rigid platform vibrating asymmetrically. Due to the spatial asymmetry and the temporal asymmetry of the system, manipulation tasks were feasible. Higashimori et al. [24,25,26] designed a system with a vibrating platform for manipulation by employing only one actuator, which was working through a mechanism containing one passive viscoelastic joint and one active joint. Manipulation of bodies on the platform was operated by regulating the shape and orientation of the spatially asymmetric vibrational orbit of the platform by tuning the offset angle and frequency of the sinusoidal excitation of the actuator. Mayyas [27,28] analyzed a vibrating platform mounted on a nonlinear leaf spring designed for manipulation based on the stick-slip motion dynamics. It was demonstrated that a required average velocity can be maintained through temporal asymmetry by tuning the amplitude and frequency of the platform excitation under the given conditions of dry friction. Mitani et al. [29,30,31,32] proposed a vibrating platform with an asymmetric microfabricated saw-tooth surface for manipulation of microparts such as multilayer ceramic capacitors. Microparts were able to move directionally due to the asymmetric adherence to the microfabricated surface. A trough with finlike asperities was used for manipulation of particles by Chen et al. [33]. The motion of the particles was caused by the force asymmetry generated by the finlike asperities as the trough was subjected to longitudinal vibrations. Liutkauskienė et al. [34] investigated a method for the manipulation of small-scale bodies on a horizontal platform subjected to vibrational excitation along the x and y directions. Different frequencies with a phase shift were excited in the x and y directions. Such sort of excitation resulted in geometrically asymmetrical oscillations of the platform, which made the bodies move. Kilikevičius et al. [35] investigated nonprehensile omnidirectional manipulation by employing a platform subjected to circular motion. The part’s motion was achieved through a frictional asymmetry created by dynamic dry friction control between the part and the platform.

In general, an asymmetry is necessary to provide and control a directional motion of a body in nonprehensile manipulation based on vibrational techniques. The scientific literature review revealed that current nonprehensile manipulation vibrational systems commonly employ temporal asymmetry, spatial asymmetry, or force asymmetry. This paper presents a novel method of nonprehensile manipulation of miniature and microminiature bodies on a harmonically oscillating platform by creating a frictional asymmetry through dynamic dry friction control. The novelty of the work is that a new type of asymmetry is proposed for a platform harmonically oscillating in the horizontal direction. This asymmetry is achieved through direct dynamic control of dry friction between the body and the platform that is implemented by periodically exciting high-frequency vibrations in the contact zone. The purpose of the presented research is to verify theoretically and experimentally the feasibility of the proposed method and determine the control parameters that define the motion characteristics, such as velocity and direction, as these are the key parameters for a manipulation system.

## 2. Methodology

### 2.1. Mathematical Model

**Figure 1:**
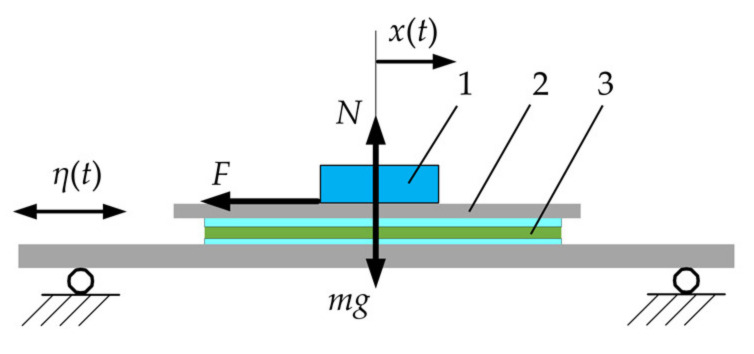
Scheme of nonprehensile manipulation on a harmonically oscillating platform with dry friction control: (1), body to be manipulated; (2), platform; (3), actuators for dry friction control.

A scheme of nonprehensile manipulation of a body on a harmonically oscillating platform with dry friction control is shown in Figure 1.

The platform is subjected to harmonic excitation along the horizontal axis:

(1) $$\eta \left(t\right)=A\;sin\;\omega t$$

where *η* is the displacement of the platform, *A* is the amplitude, *ω* is the angular frequency of the horizontal harmonic excitation, and *t* is time.

Applying D’Alembert’s principle, the relative body motion on the platform can be expressed as follows:

(2) $$\sum F-ma_{\eta }=0⟶-F-m\left(\frac{d^{2}x\left(t\right)}{dt^{2}},+,\frac{d^{2}\eta \left(t\right)}{dt^{2}}\right)=0⟶m\frac{d^{2}x\left(t\right)}{dt^{2}}-mA\omega^{2}\;sin\;\omega t+F=0$$

where *m* is the body mass, *x*(*t*) is the relative displacement of the body, and *F* is the dry friction force between the body and the platform’s upper surface. The dry friction force can be expressed as follows:

(3) $$\left\{\begin{array}{l} F=mg\mu \left(\omega t\right)\mathrm{sign}\frac{dx\left(t\right)}{dt},\;\mathrm{if}\;\frac{dx\left(t\right)}{dt}\neq 0\\ -mg\mu \left(\omega t\right)<F<mg\mu \left(\omega t\right),\;\;\mathrm{if}\;\frac{dx\left(t\right)}{dt}=0 \end{array}\right.$$

where *μ*(*ωt*) is the effective dry friction coefficient, which, in this study, is considered to be a periodic function with the same period as the horizontal harmonic oscillations of the platform *T* = 2π/*ω*.

Non-dimensionalization was applied to investigate the characteristic control parameters of the system. To non-dimensionalize Equation (2), the following were defined:

(4) $$\tau =\omega \;t,\;\xi =\frac{x\left(t\right)}{A},\;\gamma =\frac{g}{A\omega^{2}}.$$

Denoting differentiation with respect to non-dimensional time *τ* by the dot notation, the relative body motion on the platform can be expressed by the following non-dimensional differential equation:

(5) $$\ddot{\xi }=\;sin\;\tau -\gamma \mu \left(\tau \right)\mathrm{sign}\dot{\xi }.$$

The non-dimensional average velocity of the body:

(6) $$\langle \dot{\xi }\rangle =\frac{1}{2\pi }\int_{0}^{2\pi }\dot{\xi }d\tau .$$

**Figure 2:**
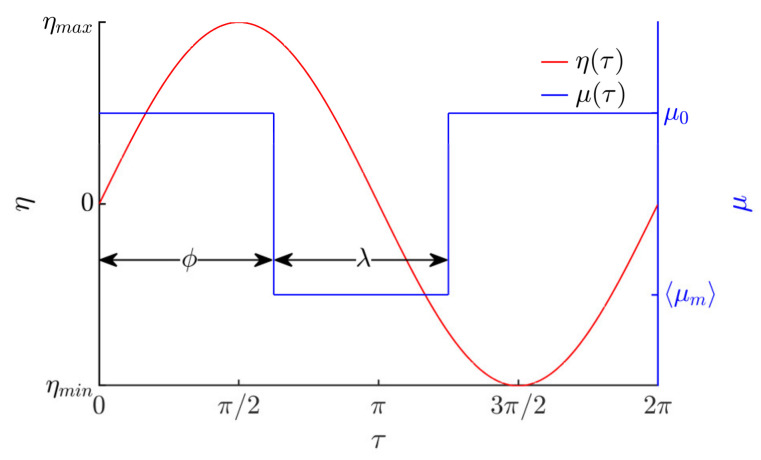
Dry friction control in respect of the period of the harmonic excitation.

In respect of the period of the harmonic excitation, the dry friction is being controlled by a rectangular function in the manner shown in Figure 2.

In respect of the period of the harmonic excitation, the rectangular function for dry friction coefficient control is expressed as follows:

(7) $$\mu \left(\tau \right)=\left\{\begin{array}{l} \langle \mu_{m}\rangle ,\mathrm{when}\;\phi +2\pi n<\tau <\phi +\lambda +2\pi n,\\ \mu_{0},\;\mathrm{for}\;\mathrm{all}\;\mathrm{other}\;\tau \;\mathrm{values}, \end{array}\right.$$

where *n* = (0, 1, 2, …), *µ*_0_ is the nominal dry friction coefficient between the body and the platform’s manipulation surface, ⟨*µ_m_*⟩ is the dynamically modified effective, time-averaged, dry friction coefficient between the body and the platform’s manipulation surface, *ϕ* is the phase shift between the horizontal harmonic excitation and the rectangular function for dry friction control, and *λ* is the width of the rectangular function.

This sort of dynamic dry friction control creates an asymmetry of frictional conditions, which is necessary to make a body move on a harmonically oscillating platform.

From the practical point of view, the effective, time-averaged friction force between the platform and the body can be dynamically controlled by exciting high-frequency vibrations in the contact zone. Many scientific papers have demonstrated that high-frequency vibrations excited in the contact zone between sliding bodies result in a reduction in the effective, time-averaged, dry friction force between the bodies due to the dynamic processes taking place in the contact zone [36,37,38,39,40]. This phenomenon can be used for dynamic dry friction control in order to create an asymmetry of frictional conditions. For example, this can be implemented by mounting actuators under the manipulation surface for high-frequency excitation in the vertical direction and periodically activating them in every period of the horizontal harmonic excitation for a fraction of *λ* with a phase shift of *ϕ*. Thus, when the actuators are activated, the effective, time-averaged, dry friction coefficient between the body and the platform’s manipulation surface becomes equal to ⟨*µ_m_*⟩, while in the remaining part of the period, the dry friction coefficient has its nominal value *µ*_0_. In this way, the dry friction force between the body and the platform can be dynamically controlled in respect of the period of the horizontal harmonic excitation according to the principle shown in Figure 2.

### 2.2. Methodology of Experimental Investigation

To test and verify that the proposed method is technically feasible and can be applied in practice for bodies of various small sizes (miniature as well as microminiature), an experimental analysis of manipulation of bodies on harmonically oscillating platforms employing dynamic dry friction control was carried out.

**Figure 3:**
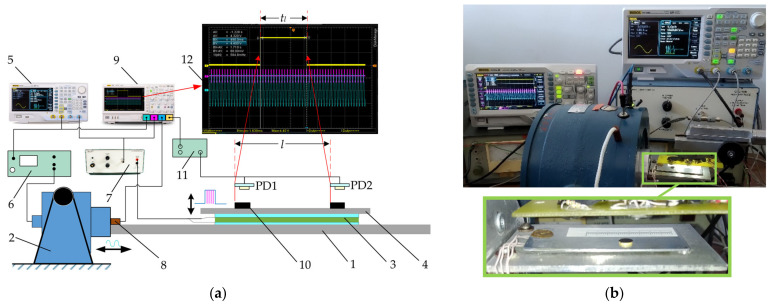
Experimental setup for manipulation of miniature bodies employing dry friction control: (**a**) principle diagram: (1), platform; (2), electrodynamic shaker; (3), piezoelectric actuator; (4), manipulation surface; (5), arbitrary waveform generator; (6), power amplifier; (7), piezo linear amplifier; (8), vibration sensor; (9), digital oscilloscope; (10), body to be manipulated; (11), photodiode signal generator; (12), view the oscilloscope screen; (**b**) general view and a zoomed view of the platform with manipulation surface.

The principle diagram of the experimental setup used for manipulations of miniature bodies is presented in Figure 3a, and a general view of the experimental setup is shown in Figure 3b. The setup consists of a horizontal platform (1), which is subjected to harmonic oscillations in the horizontal direction by using an electrodynamic shaker (ESE 211, VEB Robotron-Meßelektronik, Dresden, Germany) (2). The dynamic control of the effective friction force is implemented through a piezoelectric actuator (3) mounted on the platform. Another plate (4) is mounted on top of the piezoelectric actuator. This plate serves as a manipulation surface. The manipulation surface is polished to an average surface roughness of about 0.2 μm. When high-frequency vibrations are excited by the piezoelectric actuator, the effective friction force is reduced due to the dynamic processes taking place in the contact zone between the body and the manipulation surface. This phenomenon is used to control the dry friction force dynamically in a preferred way. An arbitrary waveform generator (DG4202, RIGOL, Beijing, China) (5) is used to generate the signals for the electrodynamic shaker and the piezoelectric actuator. The signal for the piezoelectric actuator is comprised of high-frequency (5923 Hz) pulse sequences (a duty cycle is periodically turning off and on the high-frequency excitation for the piezoelectric actuator). These pulses are synchronized with respect to the phase of the harmonic excitation in the same manner as it is presented in Figure 2. In this way, the piezoelectric actuator is being periodically activated by a rectangular function of the width of *λ* in each period of the horizontal harmonic excitation, and this function is shifted by *ϕ* from the harmonic excitation signal. During the intervals when the piezoelectric activator is active, the dry friction force between the body and the manipulation surface is reduced. Therefore, a frictional asymmetry is created by such dynamic dry friction control. The harmonic signal for the electrodynamic shaker is amplified by a power amplifier (LV-103, Metra Mess- und Frequenztechnik in Radebeul, Radebeul, Germany) (6). The signal for the piezoelectric actuator is amplified by a piezo linear amplifier (EPA-104, Piezo Systems Inc., Cambridge, MA, USA) (7). The parameters of the harmonic oscillations are monitored by a vibration sensor (8). The signals from the sensor and the generator are monitored by a digital oscilloscope (DS1054, RIGOL) (9). For the measurements of the average speed of the miniature body (*ϕ*6 × 0.8 mm, 0.201 g) (10), photodiodes (PD1 and PD2) were applied, which were connected to a photodiode signal generator (11). When the body moving on the manipulation surface crosses the photodiodes, the photodiode signal generator forms a signal, which is displayed on the screen (12) of the digital oscilloscope. The duration *t_l_* of the body’s motion between the two points is measured based on this signal. As the distance between the photodiode is *l*, the average velocity ⟨*v*⟩ = *l*/*t_l_*. This photodiode method was preferred for the measurements, which were carried out with the microminiature body since it allowed to process the results relatively fast.

**Figure 4:**
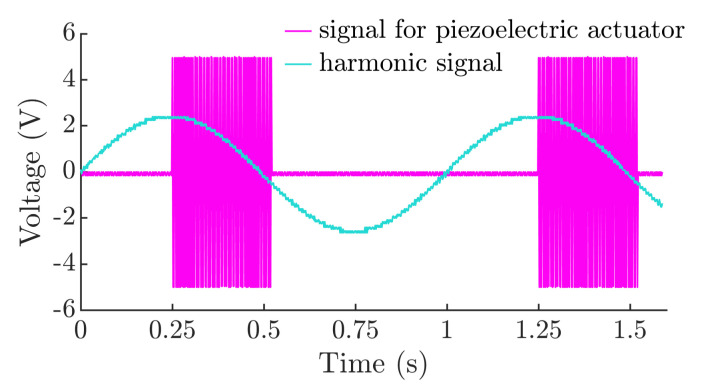
Oscillogram of the synchronized signals of piezoelectric actuator excitation and harmonic excitation when *ϕ* = 5π/9, *λ* = π/8, and *ω* = 62.83 rad/s.

An oscillogram is presented in Figure 4 that displays the synchronized signals of piezoelectric actuator excitation and harmonic excitation, which were captured by the digital oscilloscope.

**Figure 5:**
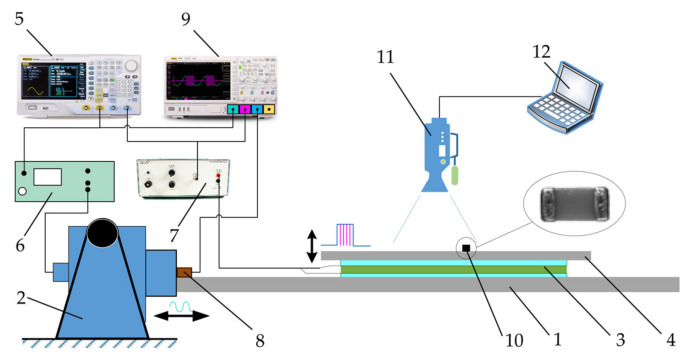
Experimental setup for manipulation of microminiature bodies employing dry friction control: (1), platform; (2), electrodynamic shaker; (3), piezoelectric actuator; (4), manipulation surface; (5), arbitrary waveform generator; (6), power amplifier; (7), piezo linear amplifier; (8), vibration sensor; (9), digital oscilloscope; (10), body to be manipulated (MLCC); (11), high-speed camera; (12), computer.

A similar approach was applied for manipulation of microminiature bodies. General multilayer ceramic capacitors (MLCC) were selected for manipulation as these microminiature bodies are widely used in microelectronics [29,30,31,32,41,42]. The developed experimental setup was tested using 0402-, 0805-, and 1206-type MLCCs (Samsung, Suwon, South Korea) (Figure 5, (10)). The principal diagram of the experimental setup used for manipulations of microminiature bodies is presented in Figure 5. In this case, the photodiode method was not able to register the average velocity of the microminiature part due to the size of the microminiature body. Therefore, digital image correlation and tracking techniques were applied for this purpose. A Phantom v711 (1280 × 800 CMOS sensor, 1 Mpx, 20 µm pixel size) high-speed camera (Vision Research, Wayne, NJ, USA) (11) was applied to record the motion of the microminiature bodies. A Canon MP-E 65 mm f/2.8 1-5× Macro lens (Canon Inc., Ōta, Tokyo, Japan) was mounted on the high-speed camera. A frame rate of 500 frames per second at a resolution of 800 × 800 was selected for the recordings. A computer (12) was used to control the camera and save the recordings. To digitize the coordinates of the microminiature body over time, a video processing program was developed in the MATLAB (MathWorks, Natick, MA, USA) programming language, employing the normalized cross-correlation method.

The experimental investigation was carried out using first-class equipment. After estimating the error of each component, the total error of the experimental results does not exceed 3%, with an exception for the measurements of the traveled distances, which were used for the calculations of the average velocity. All distance measuring scales were calibrated using a caliber of 20 mm with an error of 0.4%.

## 3. Results

### 3.1. Modeling Results

In order to determine the control parameters that define the velocity and direction of manipulation, the mathematical model was solved using the software developed in the MATLAB programing language. The Runge-Kutta ordinary differential equation solver ode45s was used to solve the non-dimensional differential motion equation.

**Figure 6:**
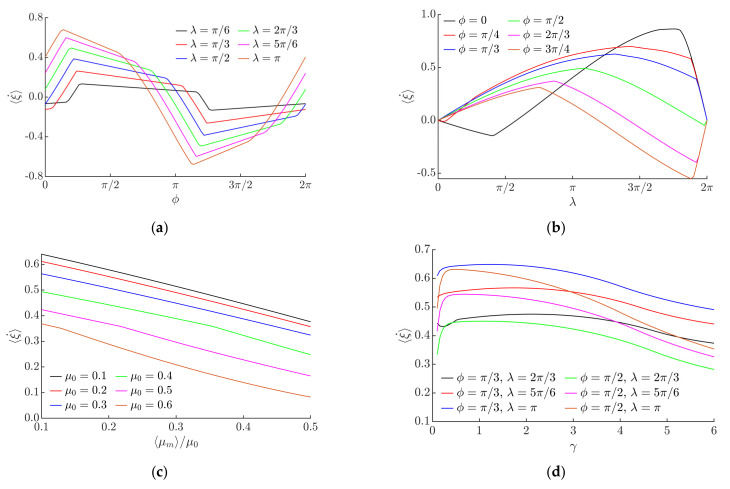
Non-dimensional average velocity depending on: (**a**) phase shift *ϕ* when *γ* = 4, *µ*_0_ = 0.1, ⟨*µ_m_*⟩/*µ*_0_ = 0.125; (**b**) *λ* when *γ* = 4; *µ*_0_ = 0.1, ⟨*µ_m_*⟩/*µ*_0_ = 0.125; (**c**) ⟨*µ_m_*⟩/*µ*_0_ when *γ* = 1, *ϕ* = π/2, *λ* = π; (**d**) *γ* when *µ*_0_ = 0.1, ⟨*µ_m_*⟩/*µ*_0_ = 0.125.

Figure 6 presents the modeling results of the non-dimensional average velocity depending on the system’s parameters. The results obviously show that the velocity and direction of the body’s motion can be controlled by changing *ϕ* (Figure 6a) and *λ* (Figure 6b). These parameters are the key control parameters defining the characteristics of the motion of the body being manipulated. Judging on the fact that the non-dimensional velocity tends to change rapidly depending on *ϕ* and reverse direction in some intervals (e.g., in an interval between approximately π and 5π/4), while, in some intervals, it tends not to change much (especially at lower *λ*, e.g., in intervals between approximately π/2 and π or between approximately 3π/2 and 2π) (Figure 6a), the phase shift *ϕ* is the parameter recommended to be used for controlling the direction of the body motion. This is due to the fact that *ϕ* defines how much the frictional asymmetry is shifted in respect of the horizontal harmonic excitation. In addition, under higher values of *λ*, the reverse of the motion direction was observed at lower values of *ϕ* (Figure 6a).

The non-dimensional velocity changes more gradually as *λ* increases. Since the parameter λ is associated with the level of the frictional asymmetry, the absolute value of the non-dimensional velocity increases when *λ* increases. Nevertheless, at some λ value, which depends on *ϕ*, it starts to decrease again as a further increase in *λ* leads to a decrease in the level of asymmetry (Figure 6b). Therefore, *λ* is the parameter recommended to be used for controlling the velocity of the body motion. When *λ* = 0 or *λ* = 2π, the non-dimensional average velocity is equal to 0 since the system is in a symmetric state, and therefore, net friction forces over one oscillation cycle cancel out. The nature of non-dimensional average velocity vs. *λ* dependencies strongly depends on *ϕ*, since this parameter defines how much the frictional asymmetry is shifted in respect of the horizontal harmonic excitation. Therefore, values of λ that result in the maximum absolute non-dimensional velocity also depend on *ϕ*, as well as *ϕ* has an influence on whether the maximum is obtained in the positive or negative direction (Figure 6b).

**Figure 7:**
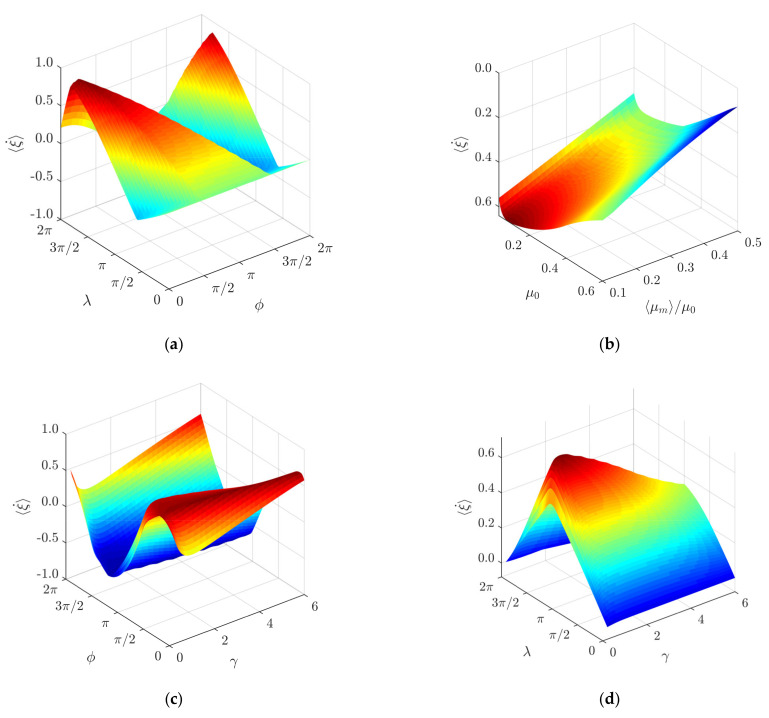
Three-dimensional representation of non-dimensional average velocity as a function of: (**a**) *ϕ* and *λ* when *γ* = 1, *µ*_0_ = 0.1, ⟨*µ_m_*⟩/*µ*_0_ = 0.125; (**b**) *µ*_0_ and ⟨*µ_m_*⟩/*µ*_0_ when *ϕ* = π*/2* *λ* = π, *γ* = 1; (**c**) *ϕ* and *γ* when *λ* = π, *µ*_0_ = 0.1, ⟨*µ_m_*⟩/*µ*_0_ = 0.125; (**d**) *λ* and *γ* when *ϕ* = π/2, *µ*_0_ = 0.1, ⟨*µ_m_*⟩/*µ*_0_ = 0.125.

A three-dimensional representation of non-dimensional average velocity as a function of *ϕ* and *λ* is presented in Figure 7a. It clearly demonstrates how the velocity and direction of the body can be manipulated by changing the phase shift *ϕ* and the width *λ* of the rectangular function for dry friction control. This theoretically verifies the feasibility of the nonprehensile manipulation method on a harmonically oscillating horizontal platform by controlling dry friction.

The modeling also revealed that the ratio of the modified effective, time-averaged, dry friction coefficient ⟨*µ_m_*⟩ to the nominal dry friction coefficient *µ*_0_ has a significant influence on the performance of the manipulation. As the ratio ⟨*µ_m_*⟩/*µ*_0_ increases, the non-dimensional average velocity decreases (Figure 6c). This is explained by the fact that lower values of ⟨*µ_m_*⟩/*µ*_0_ result in a higher frictional asymmetry. Figure 6c also suggests that the body moves slower when the nominal dry friction coefficient *µ*_0_ is higher. This is also seen from the three-dimensional representation of the non-dimensional average velocity as a function of *µ*_0_ and ⟨*µ_m_*⟩/*µ*_0_ (Figure 7b). The influence of *γ* on the non-dimensional velocity is presented in Figure 6d. The non-dimensional velocity maintains its highest values in the range of *γ* between approximately 0.25 and 3 (Figure 6d).

Since the character of the non-dimensional velocity depending on *γ* also depends on *ϕ* and *λ*, three-dimensional representations of non-dimensional average velocity as functions of *ϕ* and *γ* as well as *λ* and *γ* are presented in Figure 7c,d, respectively. It was noticed that when *γ* increases, the motion direction is reversed at slightly lower values of *ϕ* (Figure 7c).

### 3.2. Experimental Results Obtained with the Miniature Body

**Figure 8:**
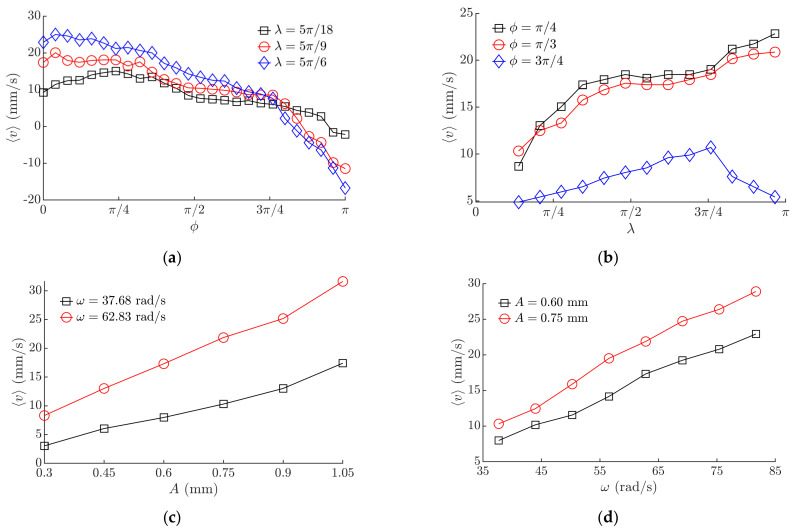
Average velocity of the miniature body depending on: (**a**) phase shift *ϕ* when *A* = 0.6 mm, *ω* = 62.83 rad/s; (**b**) *λ* when *A* = 0.6 mm, *ω* = 62.83 rad/s; (**c**) amplitude of the harmonic excitation when *ϕ* = π/6, *λ* = 4.1π/9 (**d**) angular frequency of the harmonic excitation when *ϕ* = π/6, *λ* = 4.1π/9.

The experimental investigation verified that the proposed method is technically feasible and can be applied in practice as the body was moving on the manipulation surface and responded to the tuning of *ϕ*, *λ*, and the parameters of the horizontal harmonic excitation. The experimental investigation verified the theoretical findings that the velocity and direction of the body can be controlled by changing the phase shift *ϕ* (Figure 8a) and *λ* (Figure 8b). Similar trends of the average velocity ⟨*v*⟩ depending on *ϕ* (Figure 8a) were observed as determined by the theoretical analysis (Figure 6a). Maximum values of ⟨*v*⟩ were observed in the interval of *ϕ* between 0 and π/4, and the direction of motion was reversed when *ϕ* was near π (Figure 8a). Similar trends were observed in the theoretical results as well (Figure 6a). In the analyzed interval of *λ*, at lower *ϕ* values, the average velocity ⟨*v*⟩ was increasing when *λ* was increasing (Figure 8b). When *ϕ* was 3π/4, ⟨*v*⟩ was increasing until *λ* reached 3π/4, and then started to decrease (Figure 8b). This observed nature corresponds to the theoretical findings that *λ* is associated with the level of frictional asymmetry, and this parameter defines the average velocity of the body.

The experiments also demonstrated that higher amplitudes of the harmonic excitation (Figure 8c) and higher angular frequencies (Figure 8d) result in a higher average velocity ⟨*v*⟩.

**Figure 9:**
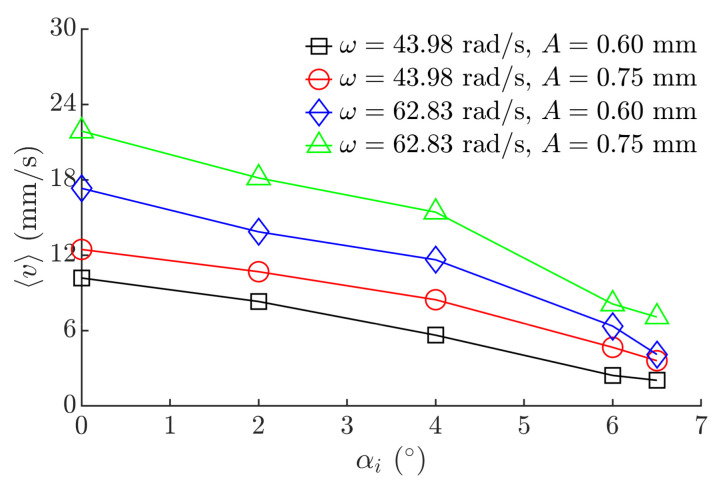
Average velocity vs. the angle of inclination of the platform when *ϕ* = π/6, *λ* = 4.1π/9.

Experiments were also carried out to identify how the angle of inclination of the platform affects the upwards motion of the miniature body. The platform was tilted with respect to the horizontal axis by *α_i,_* and the average velocity was measured. The results are presented in Figure 9. Naturally, as the angle *α_i_* of inclination of the platform was increasing, the average velocity ⟨*v*⟩ was decreasing. Nevertheless, under the investigated conditions, the body was able to maintain a stable velocity when *α_i_* was up to 6.5 degrees.

### 3.3. Experimental Results Obtained with the Microminiature Bodies

The experimental investigation carried out using the experimental setup presented in Figure 5 verified that the proposed method is also suitable for the manipulation of microminiature bodies. The microminiature bodies were moving on the manipulation surface and responded to the adjustments of *ϕ*, *λ*, and the parameters of the horizontal harmonic excitation.

**Figure 10:**
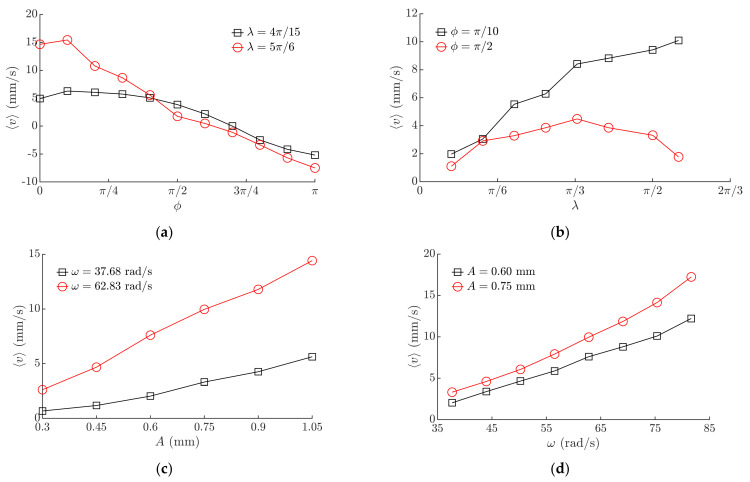
Average velocity of the microminiature body (0805) depending on: (**a**) phase shift *ϕ* when *A* = 0.6 mm, *ω* = 62.83 rad/s; (**b**) *λ* when *A* = 0.6 mm; *ω* = 62.83 rad/s; (**c**) amplitude of the harmonic excitation when *ϕ* = π/3, *λ* = 4.1π/9 (**d**) angular frequency of the harmonic excitation when *ϕ* = π/3, *λ* = 4.1π/9.

Figure 10 presents the influence of the control parameters and the harmonic excitation parameters on the average velocity of the microminiature body (0805). Maximum values of ⟨*v*⟩ were observed in the interval of *ϕ* between 0 and π/4, and the direction of motion was reversed when *ϕ* was near 3π/4 (Figure 10a). In the analyzed interval, the average velocity ⟨*v*⟩ was increasing when *λ* was increasing in the case of *ϕ* = π/2 (Figure 10b). When *ϕ* was π/2, ⟨*v*⟩ was increasing until *λ* reached around π/3, and then started to decrease (Figure 10b). The experimental results obtained with the microminiature body also indicated a similar nature of the influence of these control parameters on the motion characteristics as it was determined in the theoretical investigation (Figure 6a,b). Higher amplitudes of the harmonic excitation (Figure 10c) and higher angular frequencies (Figure 10d) result in a higher average velocity ⟨*v*⟩. Comparing these dependences to the ones obtained with the miniature body (Figure 8c,d), it is seen that values of ⟨*v*⟩ are lower in the case of the microminiature body due to different contact properties.

**Figure 11:**
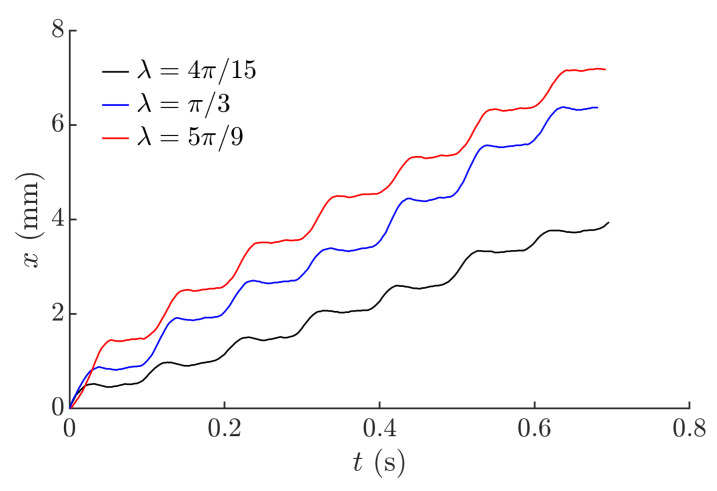
Captured trajectories of the microminiature body at different values of *λ* when *ϕ* = π/10, *A* = 0.6 mm, *ω* = 62.83 rad/s.

Captured trajectories of the microminiature body are shown in Figure 11. The moving microminiature body performs steps of a size depending on *λ*. A higher value of *λ* results in a higher step size as well. The required step size can be maintained by adjusting this parameter.

## 4. Conclusions

A novel method for the manipulation of miniature and microminiature bodies on a harmonically oscillating platform by controlling dry friction was proposed.

To theoretically verify the feasibility of the method and to determine the control parameters that define the motion characteristics, a mathematical model was developed and solved. The modeling results theoretically verified the feasibility of the nonprehensile manipulation method by demonstrating that the bodies can be moved at various velocities and the direction of motion can be changed by employing the proposed asymmetry type, which is achieved through direct dynamic control of dry friction between the bodies and the platform. The modeling revealed characteristic control parameters that define the direction and velocity of the body. It was demonstrated that the velocity and direction of the body can be controlled by changing the phase shift *ϕ* and the width *λ* of the rectangular function for dry friction control. The phase shift *ϕ* is the parameter recommended being used for controlling the direction of the body motion as this parameter defines how much the frictional asymmetry is shifted in respect of the horizontal harmonic excitation, while *λ* is the parameter recommended to be used for controlling the velocity of the body motion.

Experimental setups for miniature and microminiature bodies were developed for nonprehensile manipulation by dry friction control, and manipulation experiments were carried out. The experiments verified the theoretical findings that the direction and velocity can be controlled exactly according to the regimes determined by the control parameters *ϕ* and *λ*, and this clearly demonstrates that the proposed method is technically feasible and can be applied in practice for bodies of various small sizes (miniature as well as microminiature). The experimental investigation qualitatively verified the theoretical solution by indicating a similar nature of the influence of these control parameters on the motion characteristics as it was determined in the theoretical investigation.

The proposed method can be used in practical applications such as feeders of miniature and microminiature parts and components, handling and transportation systems, assembly lines, or other systems for manipulation of delicate components. Due to its relatively simple technological equipment and a wide range of controlled velocities, the proposed method can be used as a replacement for other more complex and expensive techniques such as manipulation devices that employ grippers. The obtained theoretical results can be used for the development of motion control algorithms and future developments of similar manipulation systems.

The presented research enriches the classical theories of manipulation methods on vibrating plates and platforms, as well as the presented results, are relevant for industries dealing with feeding, assembling, or manipulation of miniature and microminiature bodies.

## Nomenclature

*A*	Amplitude of the horizontal harmonic excitation of the platform
*a_η_*	Acceleration of the body along the horizontal axis
*F*	Dry friction force
*g*	Acceleration due to gravity
*m*	Mass of the body
*N*	Normal force
*l*	Distance between the photodiodes
*T*	Period of the harmonic oscillations of the platform
*t*	Time
*t_l_*	Duration of the body’s motion between the photodiodes
*x*	Relative displacement of the body
⟨*v*⟩	Average velocity of the body
*α_i_*	Angle between the platform and the horizontal axis
*γ*	=*g*/(*Aω*^2^)
*η*	Displacement of the platform
*λ*	Width of the rectangular function for dry friction control
*µ*	Dry friction coefficient
*µ_0_*	Nominal dry friction coefficient between the body and the platform’s manipulation surface
⟨*µ_m_*⟩	Dynamically modified effective, time-averaged, dry friction coefficient between the body and the platform’s manipulation surface
*ξ*	Non-dimensional displacement
$\langle \dot{\xi }\rangle$	Non-dimensional average velocity of the body
*τ*	Non-dimensional time
*ϕ*	Phase shift between the horizontal harmonic excitation and the rectangular function for dry friction control
*ω*	Angular frequency of the horizontal harmonic excitation
